# Genotyping and clinical characteristics of multidrug and extensively drug-resistant tuberculosis in a tertiary care tuberculosis hospital in China

**DOI:** 10.1186/1471-2334-13-315

**Published:** 2013-07-12

**Authors:** Xiaoliang Yuan, Tiantuo Zhang, Kazuyoshi Kawakami, Jiaxin Zhu, Wenzheng Zheng, Hongtao Li, Guofang Deng, Shaohua Tu, Weiyou Liu

**Affiliations:** 1 Division of Respiratory Diseases, Department of Internal Medicine, The 3rd Affiliated Hospital of Sun Yat-sen University, Institute of Respiratory Diseases of Sun Yat-Sen University, 600 Tianhe Road, Guangzhou 510630, China; 2 Department of Medical Microbiology, Mycology and Immunology, Tohoku University Graduate School of Medicine, Sendai, Miyagi 980-8575, Japan; 3 Center for Tuberculosis Diagnosis and Therapy, Jiangxi Chest Hospital, Nanchang 330006, China; 4 Department of Respiratory Medicine, The First Affiliated Hospital of Gannan Medical University, Ganzhou 341000, China

**Keywords:** *Mycobacterium tuberculosis*, Multidrug-resistant, Extensively drug-resistant, MIRU-VNTR

## Abstract

**Background:**

There is a lack of information on the clinical characteristics of multidrug-resistant (MDR) tuberculosis (TB) and extensively drug-resistant (XDR) TB in the Jiangxi Province of China; furthermore, data have not been reported on the utility of mycobacterial interspersed repetitive-unit-variable-number tandem-repeat (MIRU-VNTR) analyses in genotyping *Mycobacterium tuberculosis* strains isolated from this region. The aim of this study was to analyse the clinical features of patients with MDR and XDR TB from Jiangxi Province and to evaluate the discriminatory power of the 15-loci MIRU-VNTR method.

**Methods:**

A retrospective study was conducted on patients diagnosed with MDR and XDR TB at the Jiangxi Chest Hospital from July 2010 to June 2011. The RD105 deletion-targeted multiplex PCR (DTM-PCR) and the 15-loci MIRU-VNTR method were used to determine the genetic background of the identified MDR and XDR *M*. *tuberculosis* clinical isolates.

**Results:**

Of 804 *M*. *tuberculosis* clinical isolates, 159 (159/804, 19.8%) of the isolates were identified as MDR with first-line drug susceptibility testing. Of the 123 available MDR isolates, 13 (13/123, 10.6%) were XDR. The RD105 deletion-targeted multiplex PCR method identified 85 (85/110, 77.3%) MDR and 12 (12/13, 92.3%) XDR isolates as the Beijing genotype. MIRU-VNTR cluster analysis demonstrated that 101 MDR and 13 XDR strains had unique genotype patterns; the remaining 9 MDR strains were in 4 clusters, namely 1 cluster with 3 strains and 3 clusters with 2 strains, resulting in a low clustering rate (4.06%). The Hunter-Gaston discriminatory index (HGDI) of the 15-loci MIRU-VNTR method was as high as 0.992. In addition, clinical surveys showed that 87 (87/110, 79.1%) MDR TB patients and 10 (10/13, 76.9%) XDR TB patients had been previously treated. Diabetes mellitus was the most common comorbidity in both MDR TB (16/110, 14.5%) and XDR TB (2/13, 15.4%) patients.

**Conclusions:**

Based on our preliminary data, the MDR and XDR *M*. *tuberculosis* clinical isolates identified at the Jiangxi Chest Hospital were genetically diverse and clustered at a low frequency. The 15-loci MIRU-VNTR method showed high discriminatory power and may be used as a first-line genotyping tool in investigating the molecular epidemiology of *M*. *tuberculosis* in Jiangxi, China. Decisive measures are urgently needed to effectively prevent and manage MDR and XDR tuberculosis in this province.

## Background

The emergence of multidrug-resistant (MDR) tuberculosis (TB) and extensively drug-resistant (XDR) TB strains poses formidable challenges to the control of TB because infection with either MDR or XDR *Mycobacterium tuberculosis* strains can result in more serious disease, prolonged infectiousness, and a poor prognosis [1]. In 2010, approximately 8.8 million incident cases of TB were reported globally, and there were an estimated 650,000 prevalent cases of MDR TB [2]. According to the World Health Organization, 84 countries had reported at least 1 case of XDR TB by October 2012. China is one of the 27 high MDR and XDR TB burden countries, where the prevalence of MDR TB was found to be 5.3% and 27.4% in new and previously treated cases respectively, according to a recent systematic review and meta-analysis [3].

Determination of the clinical characteristics and molecular epidemiology of MDR and XDR TB is helpful in their diagnosis and containment. MIRU-VNTR (mycobacterial interspersed repetitive-unit-variable-number tandem-repeat) is a simpler and faster genotyping method with discriminatory power equivalent to that of IS6110 RFLP, and it has been widely used for studying the transmission dynamics of *M*. *tuberculosis*[4]. Currently, MIRU-VNTR genotyping is usually performed with one of three panels (12, 15, or 24 loci) [5,6]. Previous studies showed that the discriminatory power of MIRU-VNTR genotyping varies in different geographical areas [5,7-9]. Thus, it is necessary to choose the optimal panel or loci to improve the discriminatory power for genotyping MTB strains isolated from a given region.

Jiangxi Province is a resource-limited and high TB burden area in the southeast part of China; here, the prevalence of bacteriologically positive pulmonary TB was estimated at 203.6 cases per 100,000 inhabitants in 2000 [10]. Recently, we reported the molecular mutations and genetic lineage of MDR and XDR strains isolated from this province [10]. However, there remains little information on the clinical characteristics of MDR and XDR TB in Jiangxi Province. No study has assessed the utility of MIRU-VNTR analyses in genotyping *M*. *tuberculosis* strains isolated from this region. Therefore, we conducted a retrospective study to investigate the clinical features of patients diagnosed with MDR and XDR TB at the Jiangxi Chest Hospital and determined the genetic diversity of the identified MDR and XDR *M*. *tuberculosis* clinical isolates using the RD105 deletion-targeted multiplex PCR (DTM-PCR) and the 15-loci MIRU-VNTR method [11]. The 15-locus panel is proposed as the standard for routine epidemiological discrimination of *M*. *tuberculosis* isolates [6], the discriminatory power of the 15-loci MIRU-VNTR method used in this study was further evaluated.

## Methods

### *M*. *tuberculosis* isolates

This survey was conducted between July 2010 and June 2011 at the Jiangxi Chest Hospital located in the capital of Jiangxi Province and serving as the sole specialised tertiary care TB hospital in the province. A total of 804 *M*. *tuberculosis* clinical isolates were obtained, each from a unique patient with pulmonary TB living in Jiangxi Province. The *M*. *tuberculosis* strain H37Rv (ATCC27294) was used as a reference. All isolates were cultured in Löwenstein-Jensen medium. Species identification of *M*. *tuberculosis* was performed using *p*-nitrobenzoic acid and thiophene carboxylic acid hydrazide resistance tests [12]. This study was approved by the Institutional Review Board of the Third Affiliated Hospital of Sun Yat-Sen University and the Jiangxi Chest Hospital.

### Drug susceptibility testing

For all 804 *M*. *tuberculosis* isolates collected, first-line drug susceptibility testing (DST) was routinely performed on Löwenstein-Jensen medium using the 1% proportion method. To further identify XDR isolates, DST for four representative second-line drugs (ofloxacin, kanamycin, amikacin, and capreomycin) was performed for all available MDR strains identified in the study. This testing was performed in accordance with the WHO Guidelines [13] using the following drug concentrations: isoniazid (0.2 μg/ml), rifampin (40.0 μg/ml), streptomycin (4.0 μg/ml), ethambutol (2.0 μg/ml), ofloxacin (2. 0 μg/ml), kanamycin (30.0 μg/ml), amikacin (40.0 μg/ml), and capreomycin (40.0 μg/ml). Quality control was routinely performed during DST using the reference strain H37Rv (ATCC27294).

### Data collection and definitions

During the survey period, all MDR and XDR TB patients (n = 159) identified by DST at the Jiangxi Chest Hospital were enrolled in the study. The following clinical information was obtained from the study patients’ medical records: gender, age, complication, TB treatment history, presence of cavities on chest radiograph, and human immunodeficiency virus (HIV) status.

MDR TB was defined as a strain showing resistance to at least isoniazid and rifampicin, while XDR TB was defined as MDR TB with additional resistance to ofloxacin and at least to one of the following three second-line injectable drugs: kanamycin, amikacin, or capreomycin [14,15]. New or previously treated TB cases were defined as previously described [16].

### Genotyping procedures

Genomic DNA was extracted from *M*. *tuberculosis* isolates using the cetyltrimethylammonium bromide (CTAB) method as previously described [17]. Identification of the Beijing family was performed for all (n = 123) available MDR and XDR strains collected in this study using the RD105 deletion-targeted multiplex PCR (DTM-PCR) method [18]. All (n = 123) available MDR and XDR strains were genotyped using the 15-loci MIRU-VNTR technique as described by Supply et al. [6]. PCR products were analysed by electrophoresis on 1.5% agarose gels, and the size analysis of PCR fragments was performed using the Gel Image Analysis System (Tanon2500, Shanghai, China). The corresponding number of repetitions of various MIRU-VNTR loci of each strain was determined by comparison with the reference table from the MIRU-VNTR typing manual provided by Philip Supply (http://www.miru-vntrplus.org/MIRU/files/MIRU-VNTRtypingmanualv6.pdf). To ensure the accuracy of the DTM-PCR and MIRU-VNTR methods, the H37Rv *M*. *tuberculosis* strain and sterile water were used in each experiment as positive and negative controls, respectively.

### Cluster analysis

MIRU-VNTR genotyping data were transformed into a distance matrix on the web site MIRU-VNTR*plus* (http://www.miru-vntrplus.org) using the default setting and treated as categorical variables. Phylogenetic analysis of the distance data was conducted by the neighbour-joining (NJ) algorithm using MEGA version 5.05 [19]. A minimum spanning tree was constructed through the web site MIRU-VNTR*plus* (http://www.miru-vntrplus.org) based on the 15-loci MIRU-VNTR genotyping data [20]. The Hunter-Gaston discriminatory index (HGDI) of MIRU-VNTR types was calculated as described previously [21] and categorised according to the criteria proposed by Sola et al. [22]. Isolates with identical MIRU-VNTR genotypes were defined as belonging to the same cluster. The clustering rate was defined as (*n*_*c*_–*C*)/*N*, where *n*_*c*_ is the total number of clustered cases, *C* is the number of clusters, and *N* is the total number of cases in the sample [7].

### Statistical analysis

Categorical data analysis was performed with SPSS version 16.0 (SPSS Inc., Chicago, Illinois, USA) by using χ^2^ or Fisher’s exact tests when the theoretical frequency was less than five. A *p* value < 0.05 was considered to be statistically significant.

## Results

### Identification of MDR and XDR *M*. *tuberculosis* strains

**Figure 1:**
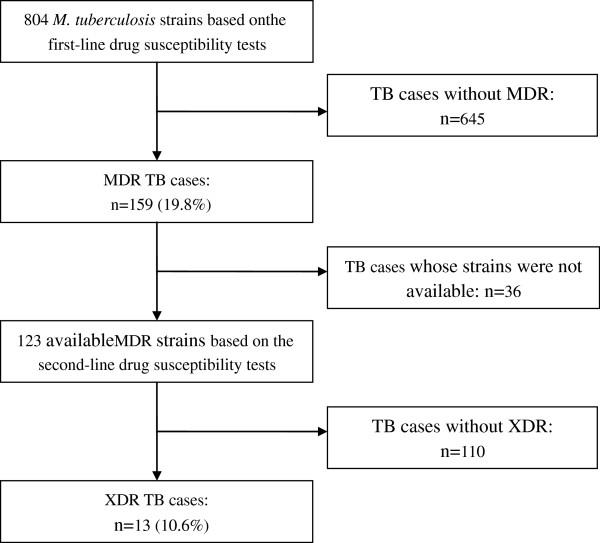
**Study profile of MDR and XDR TB patients at Jiangxi Chest Hospital, July 2010-June 2011.** TB = tuberculosis; MDR = multidrug-resistant; XDR = extensively drug-resistant.

During the study period, there were 159 (19.8%, 159/804) MDR strains identified among the 804 *M*. *tuberculosis* strains using first-line DST at the Jiangxi Chest Hospital. To determine the number and percentage of XDR strains, we performed DST using four second-line drugs on all available MDR strains in this study. However, 36 of the 159 MDR strains were contaminated, missing, or could not be recovered from storage. The remaining 123 MDR strains were tested for susceptibility to second-line drugs; of these, 13 were identified as XDR strains (10.6%, 13/123) (Figure 1).

### Clinical characteristics of MDR and XDR TB patients

We compared patient characteristics associated with MDR (n = 110) and XDR (n = 13) TB (Table 1). Eighty-six (86/110, 78.2%) of 110 MDR TB patients were 20–59 years old, and 7 (7/13, 53.8%) of 13 XDR TB cases were more likely to occur in individuals aged 40–59 years. Eighty-seven (87/110, 79.1%) MDR TB patients and 10 (10/13, 76.9%) XDR TB patients were previously treated cases (defined as patients who had received one or more months of anti-TB drugs in the past [23]). Of 123 patients, HIV status was available for 94 individuals. Among these individuals, none tested positive. It is noteworthy that diabetes mellitus was the most common comorbidity in both MDR (16/110, 14.5%) and XDR (2/13, 15.4%) TB patients. In addition, XDR TB (11/13, 84.6%) cases were more likely to present with a cavity on chest radiograph compared with MDR TB (64/110, 58.2%) cases. However, there was no significant difference in chest radiograph results between the groups.

**Table 1:** Characteristics of MDR and XDR TB patients from Jiangxi Province, China, July 2010-June 2011

**Variable**	**MDR TB, n = 110 (%)**	**XDR TB, n = 13 (%)**	**χ**^**2**^	***p*value**
Age (years)			1.61	0.81
10–19	3 (2.7)	0 (0)		
20–39	40 (36.4)	4 (30.8)		
40–59	46 (41.8)	7 (53.8)		
60–79	19 (17.3)	2 (15.4)		
≥80	2 (1.8)	0 (0)		
Sex			0.49	0.53
Male	78 (70.9)	8 (61.5)		
Female	32 (29.1)	5 (38.5)		
Cavity on chest radiograph			3.41	0.07
Yes	64 (58.2)	11 (84.6)		
No	46 (41.8)	2 (15.4)		
Treatment			0.03	1.00
New cases	23 (20.9)	3 (23.1)		
Previously treated cases	87 (79.1)	10 (76.9)		
HIV status			1.79	0.18
Positive	0 (0)	0 (0)		
Negative	86 (78.2)	8 (61.5)		
Unknown	24 (21.8)	5 (38.5)		
Comorbidity			0.00	1.00
Diabetes mellitus	16 (14.5)	2 (15.4)		
COPD	8 (7.3)	2 (15.4)		
Hepatitis	6 (5.5)	1(0.9)		
Malignancy	3 (2.7)	0 (0)		
Gout	1 (0.9)	0 (0)		
Ventricular septal defect	0 (0)	1 (7.7)		
Parkinson's disease	0 (0)	1 (7.7)		
Beijing family genotype			0.37	0.73
Yes	85 (77.3)	12 (92.3)		
No	25 (22.7)	1 (7.7)		

### Genotyping of MDR and XDR *M*. *tuberculosis* strains

**Figure 2:**
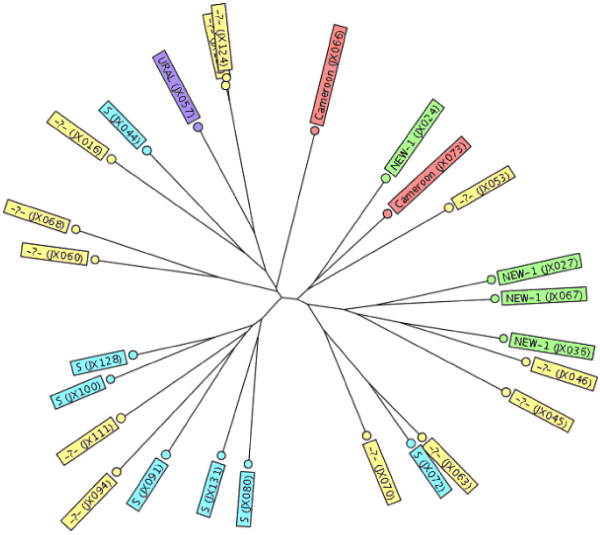
Lineage distribution of 26 non-Beijing family isolates identified by a similarity search on the MIRU-VNTRplus website.

**Figure 3:**
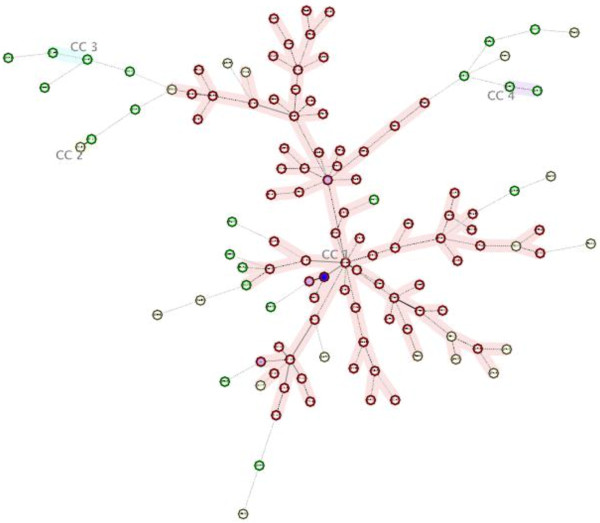
**Minimum Spanning Tree.** This tree was generated with 15 MIRU-VNTR loci of the 123 strains from Jiangxi Province, China, using the MIRU-VNTRplus database (http://www.miru-vntrplus.org). CC1, CC2, CC3, and CC4 represent four major clonal complexes. The maximum locus difference within a clonal complex is five. The red circle denotes the Beijing family strain, while the blue circle denotes the non-Beijing family strain.

We genotyped 1 isolate each from the 110 MDR TB and 13 XDR TB patients. Among them, the genetic lineage of 59 MDR strains and 10 XDR strains had been reported in a previous study [10]. Initially, the DTM-PCR method identified 85 (85/110, 77.3%) MDR and 12 (12/13, 92.3%) XDR isolates as Beijing genotype (Table 1). Furthermore, 26 non-Beijing family strains were assigned to various lineages using a similarity search on the MIRU-VNTR*plus* website. The distribution of these 26 strains within the various lineages is shown in Figure 2. The most prevalent genotype among these strains was the S family (7/26, 26.9%), followed by NEW-1(3/26, 11.5%), Cameroon (2/26, 7.7%), NEW-2 (1/26, 3.8%), and URAL (1/26, 3.8%). The remaining 12 strains (12/26, 46.2%) could not be assigned to a single lineage by the 15-loci MIRU-VNTR method. The minimum spanning tree analysis indicated that the 123 isolates were divided into 4 clonal complexes (CC1–CC4) and 22 singletons (Figure 3). CC1 included 95 strains, which mostly belonged to the Beijing family. CC2, CC3, and CC4 each contained two non-Beijing strains (Figure 3).

A dendrogram based on the 15-loci MIRU-VNTR method was constructed using a neighbour-joining algorithm and is shown in Additional file 1. Altogether, MIRU-VNTR cluster analysis showed that the 123 strains were classified into 118 genotypes. A total of 114 strains had unique patterns, while the remaining 9 strains were grouped into 4 clusters: 1 cluster with 3 strains and 3 clusters with 2 strains, resulting in a low clustering rate (4.06%). None of the XDR strains were in a genotype cluster.

**Figure 4:**
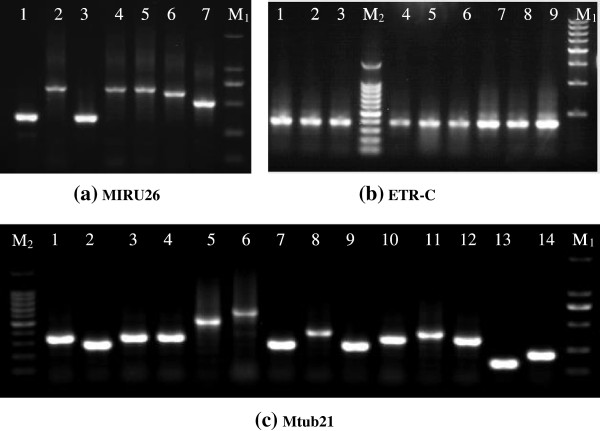
**Length polymorphisms of three loci{(a) MIRU26, (b) ETR-C, and (c) Mtub21} for some of the tested clinical isolates from Jiangxi Province, China (M**_**1**_**: 100 bp DNA Marker, M**_**2**_**: 50 bp DNA Marker).**

The allelic diversity (*h*) of the 123 *M*. *tuberculosis* strains at each MIRU-VNTR locus varied significantly, as summarised in Table 2. Among the 15 loci, the allelic diversity for 7 loci (Mtub04, Mtub21, MIRU04, MIRU26, MIRU31, QUB-11b, and QUB-26) exceeded 0.6, indicating that they are highly discriminatory. Another seven loci (MIRU10, MIRU16, MIRU40, ETR-A, QUB-4156, Mtub30, and Mtub39) showed moderate discrimination (0.3 ≤ *h* ≤ 0.6), and the final locus, ETR-C, was less polymorphic (*h* < 0.3) [22]. The highest diversity among the 123 strains was observed at MIRU26 (*h* = 0.83); the lowest diversity was observed at ETR-C (*h* = 0.02). The HGDI of the 15-loci MIRU-VNTR method was as high as 0.992. Three representative amplified bands at locus Mtub21, MIRU26, and ETR-C for some clinical strains tested are shown in Figure 4. The clinical strains demonstrated diverse electrophoretic bands at both loci Mtub21 and MIRU26, while they showed almost the same electrophoretic band at locus ETR-C.

**Table 2:** **Allelic diversity of the 15 MIRU-VNTR loci in the 110 MDR and 13 XDR *M. tuberculosis *strains from Jiangxi, China**

**VNTR locus**	**VNTR alias**	**Range of repeats**	**Allelic diversity (*h *) for**		
			**Beijing isolates (n = 97)**	**Non-Beijing isolates (n = 26)**	**All isolates (n = 123)**
424	Mtub04	1-5,7	0.49	0.74	0.61
577	ETR-C	2–4	0.01	0.04	0.02
580	MIRU04	2–8	0.75	0.67	0.75
802	MIRU40	1–4	0.33	0.62	0.43
960	MIRU10	1–6	0.34	0.65	0.49
1644	MIRU16	1–5	0.43	0.57	0.48
1955	Mtub21	0–9	0.77	0.79	0.82
2163b	QUB-11b	1–8	0.70	0.79	0.75
2165	ETR-A	1–4	0.23	0.51	0.40
2401	Mtub30	1–4	0.29	0.55	0.49
2996	MIRU26	1–10	0.79	0.79	0.83
3192	MIRU31	1–8	0.71	0.68	0.76
3690	Mtub39	1–6	0.13	0.63	0.34
4052	QUB-26	1–4,6-12	0.70	0.84	0.75
4156	QUB-4156	0-6	0.53	0.55	0.55

## Discussion and conclusions

Based on a hospital-based survey of drug-resistant cases, the findings that the rate of MDR TB was 19.8% and that 10.6% of MDR cases were XDR are similar to a report from the Chinese PLA 309 Hospital [24]. Although data from a specialised TB hospital is not representative of Jiangxi Province as a whole, our data suggest that the emergence of MDR and XDR TB may be a severe problem and that TB control is a major concern for the region. The strict Directly Observed Treatment Short Course (DOTS) is the core principle of the Stop TB Strategy and is considered to reduce the incidence of acquired drug-resistant TB [25]. The provincial project of DOTS has been implemented in the Jiangxi region since January 1, 2005. However, most of the MDR (79.1%) and XDR (76.9%) TB patients from the Jiangxi region were previously treated cases, in addition to the high rate of MDR and XDR TB cases in this study. These facts suggested that the DOTS may not be performing as well in Jiangxi Province compared with some areas of Shandong Province, which could be due to a lack of sufficient professional personnel, insufficient financial support, or patients’ poor adherence to the DOTS regimen [26]. Thus, it is necessary to adopt urgent measures for preventing and managing MDR and XDR TB effectively in this province.

In the present study, we found that most MDR and XDR TB patients were between 20 and 59 years old, with a peak frequency between 40 and 59 years old, which was in agreement with results from two recent investigations in Japan and China [27]. However, it is unclear why MDR and XDR TB were more likely to occur in individuals aged 20–59 years. This result could be partly explained by the fact that the majority of MDR and XDR TB patients undertook self-administration of treatment before the provincial DOTS project was implemented in the Jiangxi region in 2005. Inadequate monitoring and inappropriate treatment regimens could result in more drug-resistant cases than those occurring after the introduction of DOTS. Another possible explanation is poorer treatment adherence due to their heavy workloads and significant mental pressure relative to younger or older patients [28].

It is well known that diabetes mellitus is an important risk factor for TB [29]. Nevertheless, the relationship between diabetes mellitus and MDR TB is still controversial. Some studies have shown that diabetic patients are more likely to develop MDR TB compared with those without diabetes [30-32], while other studies found no significantly increased risk [33-35]. This discrepancy could be the result of these studies being hospital-based with a small sample size in different settings. A national study indicates that the age-standardised prevalence of total diabetes was 9.7% among Chinese inhabitants and that diabetes has become a major public health problem in China [36]. Diabetes mellitus is becoming the predominant force driving the spread of TB [29,31]. Thus, it is important to clarify the relationship between diabetes mellitus and drug resistance. To achieve this goal, population-based studies with large sample sizes, especially in high TB-burden countries, are urgently needed.

As anticipated, population structure analysis of MDR and XDR *M*. *tuberculosis* strains identified in this study showed that the Beijing family (97/123, 78.9%) was the predominant genotype, which is in accordance with reports from neighbouring provinces such as Anhui (85.3%), Zhejiang (70%), and Shanghai (77.1%) [7,28]. In addition, the S family (originally prevalent in Europe and America [37]) was the predominant sublineage (7/26, 26.9%) of non-Beijing family strains in this study. These results indicated that strains of the S family may have spread throughout Jiangxi Province. Further studies are required to validate this hypothesis, using more patterns of drug-resistant and drug-sensitive clinical strains.

The present study demonstrated that the discriminatory power of the 15-loci MIRU-VNTR method was very high, although the majority of the MDR and XDR strains from Jiangxi Province belonged to the Beijing family. Furthermore, 14 of the 15 loci investigated in this study (excluding ETR-C) showed relatively moderate or high discrimination (*h* > 0.3) [22]. Therefore, it can be expected that the 15-loci MIRU-VNTR technique may be a highly discriminatory tool for differentiating between clinical strains isolated from the region. However, the ETR-C locus showed very low diversity (*h* = 0.02) in this study, which is in agreement with several studies from other areas of China [4,7,38,39]. In contrast, ETR-C showed high discriminatory power in a study from Spain (*h* = 0.63) [5] and in another study from France (*h* = 0.71) [6]. These results indicate that ETR-C is not suitable for the genotyping of the *M*. *tuberculosis* strains circulating in China.

In the present study, a detailed genetic analysis of MDR and XDR *M*. *tuberculosis* isolates was performed using the MIRU-VNTR method, resulting in the identification of 123 resistant strains among patients in the Jiangxi Province of China. The 123 strains showed great genetic diversity and a low clustering percentage. In addition, most of these MDR and XDR TB patients identified at the Jiangxi Chest Hospital were previously treated cases. These results indicated that the prevalence of most MDR and XDR TB in the Jiangxi region may not be caused by clonal dissemination. Meanwhile, we noted that more than one-fifth of the MDR and XDR TB patients were new cases, which may have been caused by the active transmission of drug-resistant TB. Drug-sensitive strains are easily eliminated and their transmission is rare after treatment, whereas MDR TB is less successfully treated and spreads preferentially [40]. Therefore, primary drug resistance may also play an important role in the development of MDR and XDR TB in this region [25].

There were some limitations of the present study. First, our results likely overestimated the percentage of drug resistance compared with population-based studies because a greater proportion of seriously ill TB cases may be included in a hospital-based study. In contrast, the clustering rate of drug-resistant TB was likely underestimated in our one-year molecular epidemiologic survey because the clustering proportion depends on the completeness of sampling, where increasing sampling fractions will identify more clustering [39,41]. Second, twelve non-Beijing family isolates were categorised as “unknown” in the present study using a similarity search on the MIRU-VNTR*plus* website. This result could be due to the recent creation of the MIRU-VNTR*plus* database; the database was first published in 2008 and currently has a limited number of reference strains available for comparison [8].

Despite these limitations, we believe that the clinical and epidemiological information presented here is of particular value for the prevention and management of drug-resistant TB in the Jiangxi area. Based on our preliminary data, the MDR and XDR *M*. *tuberculosis* clinical strains identified at the Jiangxi Chest Hospital were genetically diverse and clustered with low frequency. The 15-loci MIRU-VNTR method showed a high HGDI and may be used as a first-line genotyping tool in combination with DTM-PCR for molecular epidemiological studies of *M*. *tuberculosis* in Jiangxi, China. Decisive measures are urgently needed to effectively prevent and manage MDR and XDR tuberculosis in this province.

## Competing interests

The authors declare that they have no competing interests.

## Authors’ contributions

TZ and XY participated in the conception and design of the study. ST and GD performed drug susceptibility and *M* .*tuberculosis* identification tests. HL, XY and WZ performed the PCR experiments. JZ and WL performed the acquisition of data and the data analysis. XY drafted the manuscript. KK and TZ participated in the revision of the manuscript and provided important scientific import. All authors have read and approved the final manuscript.

## Pre-publication history

The pre-publication history for this paper can be accessed here:

http://www.biomedcentral.com/1471-2334/13/315/prepub

## Supplementary Material

Additional file 1 Dendrogram of genetic relationship among the 110 MDR and 13 XDR strains from Jiangxi Chest Hospital. Click here for file
