# Supplementary material for: Genotyping and clinical characteristics of multidrug and extensively drug-resistant tuberculosis in a tertiary care tuberculosis hospital in China
Source: BMC Infect Dis. 2013 Jul 12;13:315. doi: 10.1186/1471-2334-13-315 (PMC3716566; doi:10.1186/1471-2334-13-315)
Supplement: Additional file 1 — Dendrogram of genetic relationship among the 110 MDR and 13 XDR strains from Jiangxi Chest Hospital. [file 1471-2334-13-315-S1.pdf]

Additional file 1 Dendrogram of genetic relationship among the 110 MDR and 13 XDR strains from Jiangxi Chest Hospital based on 15-locus MIRU-VNTR data

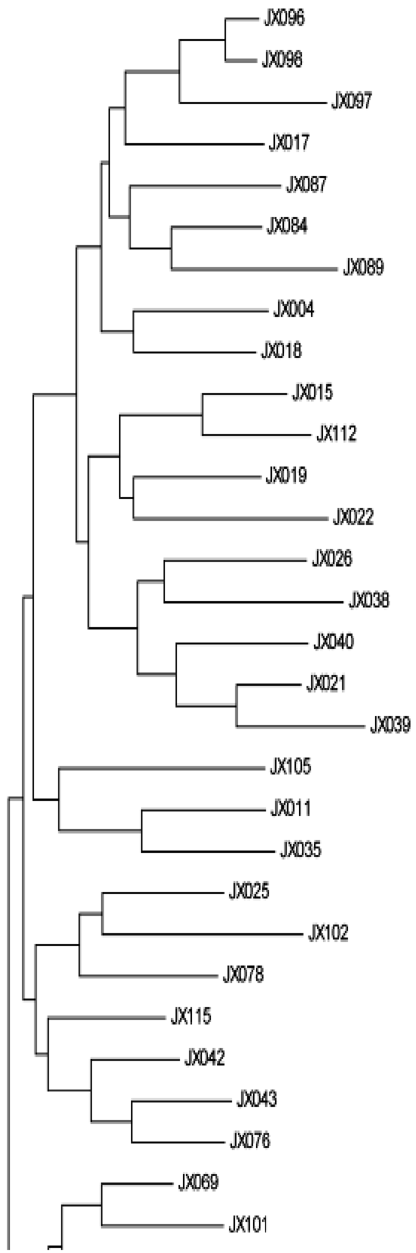

ID	424	577	580	802	960	1644	1955	2163b	2165	2401	2996	3192	3690	4052	4156
**JX096**	5	4	5	3	3	3	5	6	4	4	10	5	3	8	3
**JX098***	5	4	5	3	3	3	5	6	4	4	10	5	3	9	3
**JX097**	5	4	5	3	3	3	5	4	3	4	10	3	3	8	3
**JX017**	5	4	5	3	3	3	5	6	4	4	5	4	3	9	6
**JX087***	5	4	5	4	3	3	5	7	4	4	7	6	3	9	2
**JX084***	5	4	5	3	3	3	5	5	4	4	5	6	3	7	3
**JX089***	5	4	5	4	3	3	4	8	4	4	5	6	3	10	3
**JX004***	5	4	5	3	3	3	5	5	4	3	5	5	3	8	2
**JX018**	5	4	5	3	3	3	3	5	4	4	5	4	3	10	2
**JX015**	5	4	5	3	3	4	9	5	4	4	6	4	3	9	2
**JX112**	5	4	5	3	3	2	9	3	4	4	6	4	3	7	2
**JX019**	5	4	2	3	3	3	7	5	4	4	6	4	3	9	5
**JX022**	3	4	3	3	3	2	4	2	4	4	6	4	3	9	5
**JX026**	5	4	5	3	3	2	4	4	4	4	7	5	3	7	2
**JX038**	5	4	5	3	3	1	4	5	3	4	8	5	3	10	2
**JX040**	5	4	5	3	3	2	5	6	3	4	8	6	3	8	2
**JX021**	5	4	5	3	3	2	4	6	4	4	6	6	3	9	3
**JX039**	5	4	5	3	1	2	4	6	3	4	6	6	3	9	2
**JX105**	2	4	2	3	2	3	5	5	4	4	8	4	3	8	3
**JX011***	3	4	3	3	2	3	6	5	4	4	5	4	3	8	2
**JX035**	3	4	3	3	3	1	7	5	4	4	5	6	3	8	2
**JX025**	4	4	4	3	3	2	4	5	4	4	5	4	3	7	2
**JX102**	4	4	4	3	2	2	6	5	4	4	9	5	3	7	3
**JX078**	4	4	3	3	2	3	4	5	4	4	8	5	3	7	2
**JX115**	4	4	5	3	3	3	4	5	4	4	7	5	3	8	2
**JX042**	4	4	5	3	3	3	5	5	4	4	6	5	3	10	2
**JX043**	4	4	3	2	3	3	5	7	4	4	8	5	3	10	2
**JX076**	4	4	3	3	3	3	5	5	4	4	8	5	2	10	2
**JX069**	4	4	4	3	3	3	1	5	4	4	8	6	3	8	3
**JX101**	4	4	4	2	3	3	4	5	4	4	8	5	3	8	3

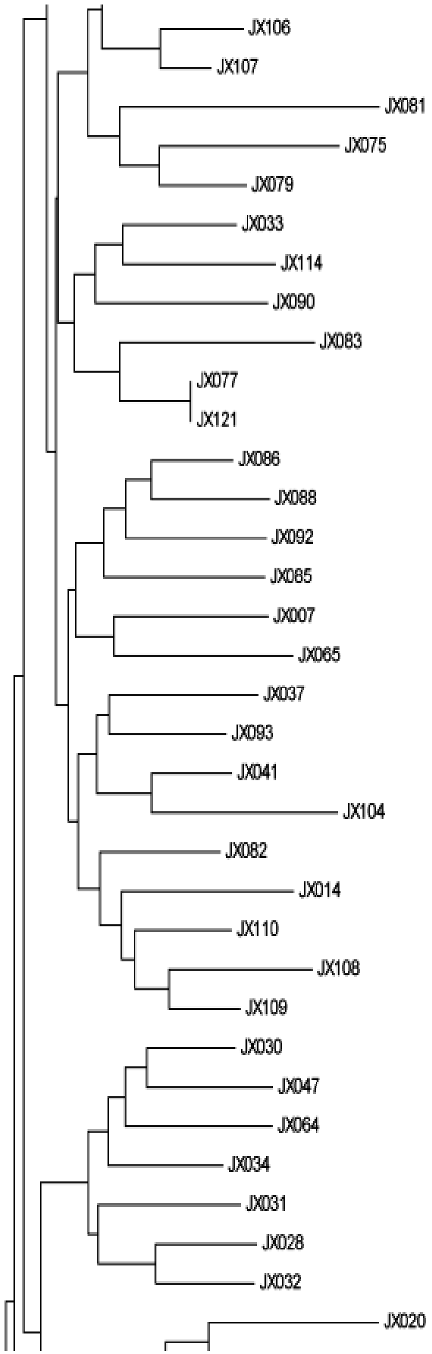

**JX106**	4	4	4	3	3	3	6	1	4	4	8	4	3	8	3
**JX107**	4	4	4	3	3	3	6	4	4	4	8	4	3	8	2
**JX081**	7	4	3	3	3	3	8	4	4	4	8	5	1	8	4
**JX075**	4	4	4	1	3	3	9	3	4	4	8	4	3	11	4
**JX079**	4	4	3	3	3	3	5	3	4	4	8	4	3	8	4
**JX033**	4	4	4	3	3	1	5	5	4	4	6	6	3	8	2
**JX114**	4	4	3	3	3	5	5	3	4	4	6	4	3	8	2
**JX090***	4	4	4	4	3	3	5	4	4	4	4	6	3	8	2
**JX083**	3	3	3	3	3	3	5	7	4	4	8	6	3	9	2
**JX077**	4	4	3	3	3	3	5	6	4	4	8	6	3	8	2
**JX121**	4	4	3	3	3	3	5	6	4	4	8	6	3	8	2
**JX086***	4	4	4	3	3	3	8	7	4	4	7	6	3	9	2
**JX088***	4	4	4	4	3	3	2	5	4	4	7	6	3	9	2
**JX092**	4	4	4	3	3	3	4	5	4	4	7	7	3	9	4
**JX085***	4	4	4	3	3	3	7	3	4	4	6	6	3	9	3
**JX007***	4	4	4	3	3	3	6	5	4	3	6	5	3	9	2
**JX065**	4	4	4	3	4	3	3	5	4	3	8	6	3	8	2
**JX037**	4	4	4	3	3	1	7	6	4	4	8	4	3	9	2
**JX093**	4	4	4	3	3	3	5	6	4	4	10	8	3	9	2
**JX041**	4	4	4	3	3	3	5	5	3	4	8	6	3	9	2
**JX104***	4	4	4	3	5	3	5	6	3	4	8	3	3	12	2
**JX082**	4	4	4	3	3	3	3	4	4	4	8	5	3	9	2
**JX014**	4	4	6	3	3	3	2	4	4	4	3	4	3	10	2
**JX110**	4	4	4	3	3	3	4	4	4	4	1	4	3	9	2
**JX108**	5	4	3	3	3	3	5	4	4	4	8	4	5	9	2
**JX109**	4	4	3	3	3	3	5	4	4	4	1	4	3	9	
**JX030**	4	4	2	3	4		5	4	4	4	6	6	3	9	2
**JX047**	4	4	2	3	4	4	5	6	4	4	7	3	3	9	2
**JX064**	4	4	2	3	4	4	5	6	4	4	8	6	3	8	3
**JX034**	4	4	2	3	4	3	6	6	4	4	6	6	3	8	2
**JX031**	4	4	4	3	4	3	6	6	4	4	7	6	3	9	3
**JX028**	4	4	4	3	4	2	5	6	4	4	6	8	3	9	2
**JX032**	4	4	4	3	4	2	6	5	4	4	6	6	3	9	2
**JX020**	4	4	2	1	4	2	3	6	4	4	6	4	3	9	4

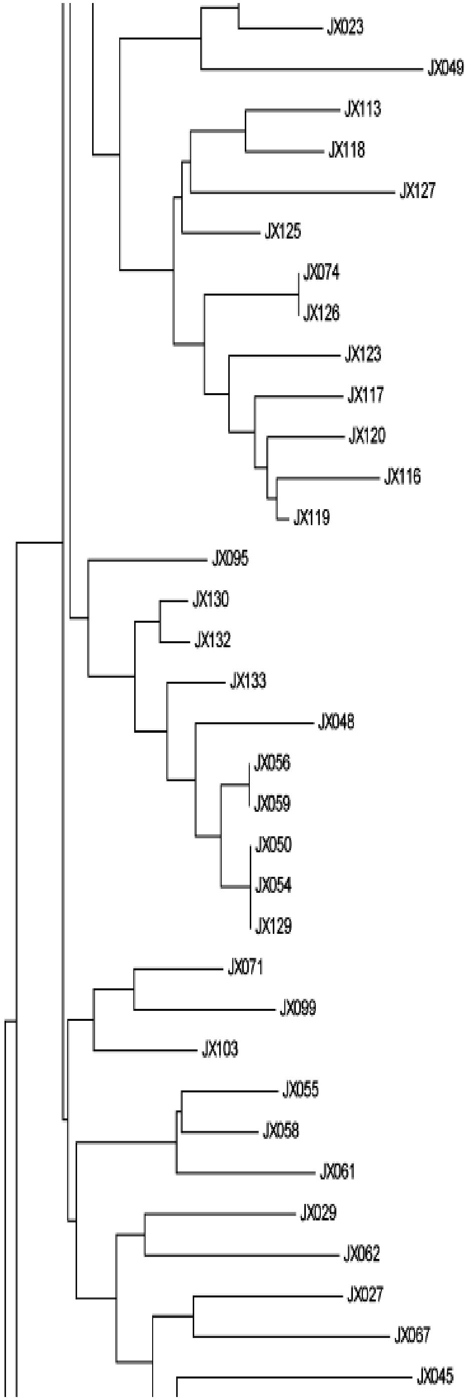

**JX023**	5	4	2	3	3	2	4	6	4	4	6	4	3	9	4
**JX049**	2	4	2	3	4	3	4	6	4	3	7	4	5	9	4
**JX113**	5	4	3	2	3	2	4	6	4	4	3	4	3	8	2
**JX118**	4	4	3	2	3	2	4	6	4	4	7	4	3	11	2
**JX127**	4	4	3	1	3	4	4	6	1	4	7	4	3	9	0
**JX125**	4	4	2	2	3	3	4	6	4	4	7	4	3	9	2
**JX074**	4	4	3	2	2	3	5	6	4	4	7	4	3	8	2
**JX126**	4	4	3	2	2	3	5	6	4	4	7	4	3	8	2
**JX123**	4	4	3	2	3	3	5	6	1	4	7	3	3	6	2
**JX117**	4	4	3	2	3	3	3	6	2	4	7	4	3	7	2
**JX120**	4	4	3	2	3	3	7	6	1	4	7	4	3	10	2
**JX116**	4	4	3	2	3	3	4	6	2	4	7	4	5	10	2
**JX119**	4	4	3	2	3	3	4	6	4	4	7	4	3	10	2
**JX095**	4	4	2	3	3	3	4	7	4	4	9	6	3	8	2
**JX130**	4	4	2	3	3	3	5	6	4	4	7	5	3	8	2
**JX132**	4	4	2	3	3	3	5	6	4	4	5	5	3	8	2
**JX133**	4	4	2	3	3	3	5	6	4	3	7	6	3	8	2
**JX048**	4	4	2	3	4	5	4	6	4	3	7	5	3	8	2
**JX056**	4	4	2	3	3	3	1	6	4	3	7	5	3	8	2
**JX059**	4	4	2	3	3	3	1	6	4	3	7	5	3	8	2
**JX050**	4	4	2	3	3	3	9	6	4	3	7	5	3	8	2
**JX054**	4	4	2	3	3	3	9	6	4	3	7	5	3	8	2
**JX129**	4	4	2	3	3	3	9	6	4	3	7	5	3	8	2
**JX071**	4	4	2	3	3	3	4	6	4	4	9	6	3	7	3
**JX099**	2	4	2	3	3	3	4	6	4	4	10	5	3	2	3
**JX103**	4	4	2	3	3	3	5	5	4	4	9	5	3	9	3
**JX055**	3	4	3	3	3	3	6	5	4	3	7	6	3	9	3
**JX058**	3	4	2	3	3	3	5	6	4	3	7	6	3	9	3
**JX061**	3	4	2	3	3	5	9	5	4	3	8	6	3	9	3
**JX029**	4	4	2	3	4	3	4	7	3	4	5	6	3	9	3
**JX062**	5	4	2	3	3	3	4	3	3	3	8	6	3	6	3
**JX027**	3	4	2	3	2	3	2	3	3	4	6	4	3	10	2
**JX067**	3	4	2	3	4	3	3	3	3	2	8	1	3	7	2
**JX045**	2	4	2	2	6	3	2	1	3	4	3	5	3	4	2

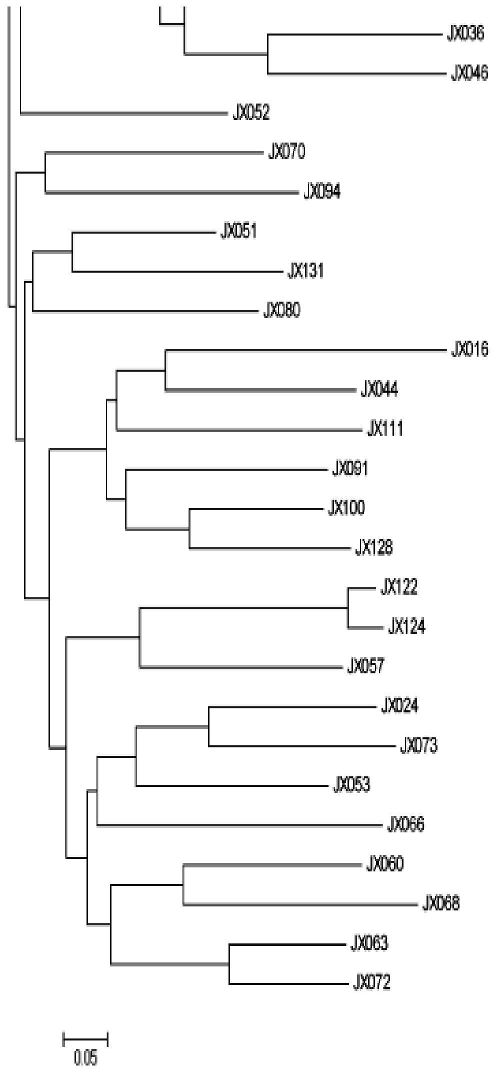

**JX036**	2	4	2	3	1	1	4	2	3	2	5	5	3	7	2
**JX046**	2	4	2	3	3	5	3	2	3	3	5	5	3	6	1
**JX052**	4	4	2	3	3	3	3	6	4	1	4	6	1	8	3
**JX070**	4	4	2	3	2	3	6	3	4	2	6	4	1	6	3
**JX094**	4	2	7	3	3	3	1	2	4	2	9	5	1	6	2
**JX051**	4	4	4	3	3	3	1	5	4	1	5	3	1	8	3
**JX131**	4	4	3	2	3	3	1	5	3	2	5	2	1	8	2
**JX080**	4	4	8	2	2	3	6	2	4	2	5	3	3	8	2
**JX016**	5	4	4	2	2	4	0	4	3	1	4	3	1	10	3
**JX044**	5	4	3	2	2	2	0	2	4	3	6	3	1	9	2
**JX111**	5	4	6	3	2	2	1	3	4	2	4	2	1	1	2
**JX091**	2	4	7	2	3	3	1	3	4	3	4	3	1	7	2
**JX100**	2	4	5	1	2	3	1	6	4	2	7	3	1	9	2
**JX128***	4	4	5	2	2	3	1	6	3	2	4	3	1	4	2
**JX122**	3	4	2	2	5	2	3	5		4	2	3	4	9	3
**JX124**	3	4	2	1	5	2	3	5		4	2	3	4	9	3
**JX057**	3	4	3	3	2	2	3	5	4	1	1	3	1	8	3
**JX024**	1	4	2	3	2	3	1	3	3	2	5	3	1	7	4
**JX073**	2	4	2	3	2	2	6	2	3	2	5	3	1	4	3
**JX053**	1	4	2	3	3	3	6	2	3	1	4	3	2	9	3
**JX066**	3	4	2	2	3	2	4	4	3	2	4	3	6	5	2
**JX060**	3	4	3	1	3	2	6	6	3	2	5	6	1	9	3
**JX068**	2	4	3	2	4	2	5	6	3	2	5	2	5	6	3
**JX063**	3	4	2	1	2	3	6	4	3	2	6	6	3	3	3
**JX072**	3	4	3	1	2	3	1	4	3	2	6	4	3	6	3

Note: * indicates XDR *M. tuberculosis* strain.
